# Conservative Management of Abnormally Invasive Placenta Previa after Midtrimester Foetal Demise

**DOI:** 10.1155/2018/7478437

**Published:** 2018-10-14

**Authors:** A. MacGibbon, Y. M. Ius

**Affiliations:** Department of Obstetrics and Gynaecology, John Hunter Hospital. Newcastle, New South Wales, Australia

## Abstract

We present the case of a midtrimester intrauterine foetal demise (IUFD) in the context of abnormally invasive placentation. This was a grade 4 placenta previa with placenta increta in a patient requesting fertility conservation and was managed conservatively without immediate surgical intervention. The patient spontaneously delivered the fetus after 33 days, followed by a large obstetric haemorrhage requiring immediate laparotomy and hysterotomy. Her uterus was preserved and she went on to recover without further significant complication. While conservative management of morbidly adherent placentas has been well documented, there are no published cases of this strategy in the context of IUFD and fertility preservation.

## 1. Introduction

A morbidly adherent placenta poses a significant morbidity and mortality risk to pregnant women, primarily due to major haemorrhage at time of delivery. This includes placenta accreta where the placenta attaches to the myometrium, increta where it penetrates through the myometrium, and percreta where it invades surrounding structures such as bladder or bowel. Generally, these cases are managed by elective caesarean hysterectomy due to the risks associated with attempted removal of the placenta alone. This comes with the unfortunate consequence of loss of fertility for the patient. Of particular interest, therefore, are any strategies that can safely and effectively manage both the major praevia and the morbidly adherent placenta while providing the opportunity to preserve the uterus.

Nonsurgical strategies have effectively preserved fertility in many cases [1–3]; however no studies have yet examined the safety and efficacy of this management strategy in the complicated context of foetal demise. We present the case of a midtrimester intrauterine foetal demise in a patient with a morbidly adherent placenta and grade 4 praevia.

## 2. Case Description

A 31-year-old gravida 3 para 1 patient presented to antenatal clinic at 19 weeks and 3 days' gestation to discuss the results of her morphology scan which had demonstrated a grade 4 placenta previa covering the cervical os. She had a medical history significant for Arnold Chiari malformation requiring craniotomy in 2006 as well as correction of a Syringomyelia in 2005. She also suffers from irritable bowel syndrome but was taking no regular medications and had a BMI of 23. Her first pregnancy resulted in a spontaneous miscarriage that did not require dilatation and curettage. Her second pregnancy resulted in a planned elective caesarean due to concerns about raised intracranial pressure during labour, as recommended by her neurologist. She had routine antenatal care this pregnancy which had been unremarkable to date.

The morphology scan demonstrated a small omphalocele but otherwise no significant structural defects and estimated foetal weight was noted to be within the normal range. During the clinic review, the fetus was found to have a heart rate well below 100 bpm. Repeat ultrasound the following day confirmed IUFD. This ultrasound also demonstrated evidence of an abnormally invasive placenta with the appearance of dysplastic vascular hypertrophy. An obstetric MRI was performed which supported the diagnosis of morbidly adherent placenta. This showed a low lying inhomogeneous placenta, dysplastic vascular hypertrophy, ill-defined placental bands, and an overall impression of some areas of increta with no overt evidence of percreta.

Options were discussed with the patient who decided for conservative management in order to optimise her chance of preserving her fertility. This was balanced against potential complications of prolonged conservative management of an IUFD, including sepsis and coagulopathy. A plan was made for serial ultrasounds as an outpatient, to be followed by induction of labour when placental blood flow was no longer detectable. Twenty-seven days following IUFD confirmation, the patient was admitted to hospital with abdominal cramping and associated small antepartum haemorrhage (APH).

Ultrasound scan at 31 days showed a minimal reduction in blood flow through the anterior placenta and to the cervix. At day 33 she suffered a further 300mL APH. Given her increasing blood loss and minimal changes to placental blood flow on ultrasound, she was administered a dose of 80mg methotrexate intramuscularly with the hope of accelerating devitalisation of the placenta. A repeat dose of methotrexate was planned for five days' time. During the subsequent two days after the administration of methotrexate, the patient continued to suffer moderate bleeds and increasingly significant contractions. 35 days following IUFD she spontaneously delivered a male fetus with only minimal bleeding during delivery.

A brisk 2 L postpartum haemorrhage (PPH) followed delivery and the patient was immediately taken to the operating theatre for examination under anaesthesia and attempted manual removal. A urinary indwelling catheter was inserted and remained in situ for the entirety of the operation. Due to only partial removal (approximately 80%) of the placenta being achieved manually, the case quickly progressed to laparotomy. Intraoperative findings revealed a full thickness increta at the previous caesarean incision just above the level of the bladder. Hysterotomy was performed with a transverse incision made above the prior caesarean incision, and the remaining placenta was removed manually, creating a 3x3cm plug-like defect anteriorly. This defect was closed with a primary closure separate to the hysterotomy incision. In addition, the placental bed was oversewn to establish haemostasis. A Foley's catheter was inserted vaginally and inflated with 60mL normal saline. Total blood loss was 4 litres (L): 2L immediately postpartum, 1L while attempting per vaginal manual removal of the placenta, and 1L intraoperatively. Massive transfusion protocol was activated with the patient receiving 10 units of packed red cells, 6 units of fresh frozen plasma and 5 units of cryoprecipitate. The patient remained stable throughout the process. A further 3 units of packed cells was given over the next two days for persistent anaemia. The fetus was found to weigh 170g. No cause for foetal demise was identified and the family decided against an autopsy. Pathological examination of the placenta was performed. This was noted to be difficult due to extensive haemorrhage and areas of necrosis commensurate with intrauterine foetal death and prolonged intrauterine retention. There was no evidence of funisitis or umbilical cord vasculitis to support a diagnosis of chorioamnionitis nor were any pathogens observed. The degree of decidual haemorrhage and necrosis made a histological diagnosis of placenta accreta impossible.

The patient recovered without significant complication over the following days and was discharged 1 week later on oral antibiotics and aperients. Six weeks after discharge the patient was seen in a postnatal follow-up clinic. She experienced minimal lochia in the postpartum period and was feeling generally well.

## 3. Discussion

Generally foetal loss in the second or third trimester is managed by either dilatation and curettage or induction of labour depending on the gestation. However, in the case of a morbidly adherent placenta, this presents a large risk of uncontrollable haemorrhage. Furthermore placenta previa increases the risk of complications as a vaginal delivery requires the placenta to deliver prior to, or simultaneously with the fetus. In this uncommon and difficult clinical scenario, the morbidly adherent placenta previa blocks the delivery of the fetus through the cervical canal. The generally accepted management strategy for abnormally adherent placentation is caesarean hysterectomy [4, 5]. However, since the 1950s, conservative treatments have been described for accreta and increta with the aim of preserving the uterus and thereby the fertility of these women [6]. This often involves leaving the placenta in situ and waiting for devascularisation of the placental bed so that remaining placental tissue may either be more safely removed or resorb itself. While there is a high rate of recurrence of placenta accreta (17-29%), several studies have reported excellent fertility rates following conservative treatment [2, 3].

Treating an abnormally adherent placenta conservatively must be weighed against the risk of several associated complications. The most significant of these is ongoing bleeding or secondary postpartum haemorrhage. A third of women being treated conservatively are likely to suffer ongoing vaginal bleeding—in the study by Timmermans et al., 15% of such patients went on to require delayed hysterectomy [1]. In a study by Sentilhes et al., 42% of women required a blood transfusion while being managed conservatively and 15% required more than 5 units [7]. Infection is another concern, however it may be difficult to diagnose clinically due to fever being a result of tissue necrosis alone in some cases [1]. Endometritis was diagnosed in 18% of patients in the Timmermans study, 18% of whom required hysterectomy as a result [1]. It must be noted however that infection rates amongst women with an IUFD and no rupture of membranes may well be lower. A further well-documented concern amongst patients conservatively managing IUFD is that of disseminated intravascular coagulopathy (DIC). While waiting for an abnormally adherent placenta to devascularise or resorb can take several months [1, 7]; the risk of DIC is up to 10% within 4 weeks of foetal death and continues to increase after this time [8]. Weighing the risk of these complications against the immediate loss of fertility from hysterectomy without clear guidelines presents an ongoing challenge for clinicians.

### 3.1. Monitoring of These Patients

In this presented case, the patient was initially diagnosed with placenta increta using ultrasound and subsequently MRI. She was then monitored with serial ultrasound looking for devascularisation of the placental bed. Ultrasound is an effective imaging modality for diagnosing and monitoring placental invasion [1]. There has been no consistently demonstrated superiority in sensitivity or specificity of MRI over serial ultrasound in the diagnosis of morbidly adherent placenta. However, the use of both techniques may be useful to evaluate the extent of placental tissue invasion [5, 9]. HCG has previously been suggested as a method of monitoring placental vascular activity [10] but there are several documented cases of persistent vascularity despite undetectable HCG levels making it an unreliable marker [1].

### 3.2. Methotrexate

Since the 1980s methotrexate has been suggested as an adjuvant treatment during the observation period in order to target folate metabolism in the rapidly growing tissues of the trophoblast [11]. There is currently no clear evidence as to the benefit of methotrexate in conservative management of placenta accreta after delivery [12]. This has been attributed to limited or absent trophoblastic proliferation at term [13] and as such is not recommended by UK or American guidelines [4, 5]. In the case of IUFD or midtrimester miscarriage, however, the much higher rate of proliferation could make methotrexate a more effective treatment. Consideration must also be given to the adverse effects of methotrexate which range from nausea, headache, and fevers to nephrotoxicity and rarely severe myelosuppression that can be fatal [7].

### 3.3. Learnings from This Case

The aim of conservative management of abnormally invasive placentation is to allow the placenta time to devitalise and hopefully make removal less difficult. While this case resulted in a 4L PPH and surgical removal of the invasive placental tissue, the uterus was ultimately preserved. If delivery and removal of the placenta had been attempted at time of diagnosis bleeding from the placental bed may well have been more difficult to control leading to a hysterectomy. Both UK and American guidelines are clear that these women must be managed in a centre capable of rapid blood transfusions, with emergency operating theatres, experienced multidisciplinary surgeons, and intensive care [4, 5]. This is well supported by the outcome of this case. Other significant risks during the observation period include infection, ongoing bleeding, and DIC along with what may be a traumatic psychological experience for the woman [1, 14]. Placental vascularity has been shown to persist for months in some cases [1] and in many women; having a retained fetus for this length of time would not be acceptable. Despite these risks, conservative management will continue to be a desirable option for women wanting to preserve their fertility, particularly in the context of IUFD. With the number of caesarean deliveries increasing worldwide, this complex clinical scenario is likely to become more common [15]. Further investigation into the best markers for placental vascularity and possible benefits of methotrexate in midtrimester loss would be helpful to develop an appropriate management protocol for these patients.
